# Influence of Different Conditioning Treatments on the Bond Integrity of Root Dentin to rGO Infiltrated Dentin Adhesive. SEM, EDX, FTIR and MicroRaman Study

**DOI:** 10.3390/polym13101555

**Published:** 2021-05-12

**Authors:** Firas K. Alqarawi, Mazen F. Alkahtany, Khalid H. Almadi, Afnan A. Ben Gassem, Faris A. Alshahrani, Mohammad H. AlRefeai, Imran Farooq, Fahim Vohra, Tariq Abduljabbar

**Affiliations:** 1 Department of Substitutive Dental Sciences, College of Dentistry, Imam Abdulrahman Bin Faisal University, Dammam 34212, Saudi Arabia; fkalqarawi@iau.edu.sa (F.K.A.); faalshahrani@iau.edu.sa (F.A.A.); 2 Department of Restorative Dental Science, Division of Endodontics, College of Dentistry, King Saud University, Riyadh 11545, Saudi Arabia; malkahtany@ksu.edu.sa (M.F.A.); kalmadi@ksu.edu.sa (K.H.A.); 3 Department of Pediatric Dentistry and Orthodontics, College of Dentistry, Taibah University, Al-Madinah, Al-Munawwarah 42353, Saudi Arabia; A_bengassem@hotmail.com; 4 Department of Restorative Dentistry, Division of Operative Dentistry, King Saud University, Riyadh 11545, Saudi Arabia; malrefeai@ksu.edu.sa; 5 Faculty of Dentistry, University of Toronto, Toronto, ON M5G 1G6, Canada; imran.farooq@mail.utoronto.ca; 6 Department of Prosthetic Dental Science, College of Dentistry, King Saud University, Research Chair for Biological Research in Dental Health, College of Dentistry, Riyadh 11545, Saudi Arabia; tajabbar@ksu.edu.sa

**Keywords:** adhesive, graphene, rGO, NaOCl, PDT, ECYL

## Abstract

The present study aimed to synthesize and equate the mechanical properties and dentin interaction of two adhesives; experimental adhesive (EA) and 5 wt.% reduced graphene oxide rGO) containing adhesive. Scanning electron microscopy (SEM)-Energy-dispersive X-ray spectroscopy (EDX), Micro-Raman spectroscopy, push-out bond strength test, and Fourier Transform Infrared (FTIR) spectroscopy were employed to study nano-bond strength, degree of conversion (DC), and adhesive-dentin interaction. The EA was prepared, and rGO particles were added to produce two adhesive groups, EA-rGO-0% (control) and rGO-5%. The canals of sixty roots were shaped and prepared, and fiber posts were cemented. The specimens were further alienated into groups based on the root canal disinfection technique, including 2.5% sodium hypochlorite (NaOCl), Photodynamic therapy (PDT), and ER-CR-YSGG laser (ECYL). The rGO nanoparticles were flake-shaped, and EDX confirmed the presence of carbon (C). Micro-Raman spectroscopy revealed distinct peaks for graphene. Push-out bond strength test demonstrated highest values for the EA-rGO-0% group after NaOCl and PDT conditioning whereas, rGO-5% showed higher values after ECYL conditioning. EA-rGO-0% presented greater DC than rGO-5% adhesive. The rGO-5% adhesive demonstrated comparable push-out bond strength and rheological properties to the controls. The rGO-5% demonstrated acceptable DC (although lower than control group), appropriate dentin interaction, and resin tag establishment.

## 1. Introduction

Dental caries is a multifactorial infectious disease that results in tooth structure loss [1]. To restore caries that have not yet reached the pulp, various restorative materials, including dental amalgam, dental composites, and glass-ionomer cements are used [2]. Among these materials, dental composites are the most popular, primarily due to their superior aesthetics [3]. Dental composites are placed in the oral cavity, which is an exceptionally challenging environment—exposure to temperature fluctuations, moistness, and abrasion offered by the toothbrushes, all have to be dealt with [4]. Dentin adhesives play a pivotal role in influencing the longevity of dental resin composites [5]. A strong adhesive-dentin bond ensures that the resin composite will endure a harsh oral environment and masticatory challenges [6]. These adhesives are used to bond the hydrophilic dentin with hydrophobic resin composites [7]. In general, adhesion with dentin is more challenging than its neighboring enamel, as the former is more organic with greater water content than the latter, requiring more technique sensitivity [8]. The adhesive bond’s quality depends on the competence of monomers to invade spaces between collagen fibers of dentin and form steady resin tags to create a hybrid layer [9]. An unstable adhesive bond loses strength over time, leading to an eventual restorative failure [10]. To counteract this loss of adhesive’s bond strength, inorganic fillers are added to the adhesive that improves their dentin interaction and probably curtail the degradation of the adhesive [11]. Past studies have validated that nanofillers’ incorporation in the adhesive reduces their solubility and improves their mechanical properties [12,13]. Accordingly, the inclusion of nano-inorganic fillers to enhance various properties of dentin adhesives is warranted.

Graphene-based materials (GBMs) have recently caught the attention of dental researchers. The GBMs are thermally and chemically stable and enjoy a high surface area [14]. Among the GBMs, graphene oxide (GO) is a unique material that can be obtained by the oxidation of graphite [15]. Although graphene is a hydrophobic material, GO is considered hydrophilic as it contains oxygen in its functional groups [16]. This hydrophilic nature of GO is beneficial as it assists it in developing steady colloid dispersal and becomes cytocompatible [17]. Mei et al. in an earlier study verified that the addition of GO-silica particles could enhance the compressive strength of experimental adhesives [18]. Haeri et al. reported a significant increase in the mechanical properties of composites which were reinforced with GO sheets and silica nanoparticles [19]. In another similar study, Ozcan et al. also reported significant improvements in the material, brought by the addition of silica and GO nanohybrid particles [20]. Although reduced GO (rGO) has less functional groups compared with GO, its *sp*^2^ structures and physical properties are still recoverable, and it is also purer and contains more graphene properties than GO [21]. Studies that have incorporated GO as a filler in the adhesive are abundant; however, limited data is available when it comes to the studies that have checked the efficacy of rGO filler particles in improving the adhesives’ mechanical properties. Even though these studies are scarce in the literature, the use of rGO has been advocated as its inclusion could improve dental composites’ mechanical and rheological properties [22,23]. 

Considering the beneficial properties of rGO, it can be postulated that their inclusion could improve dentin adhesives’ mechanical and rheological properties. Henceforth, the present study was aimed at developing an experimental adhesive (EA) containing rGO nanoparticles. It was hypothesized that the inclusion of rGO nanoparticles would improve dentin interaction, durability, bond strength, and rheological properties of the adhesive. Our study was directed at synthesizing and characterizing an EA containing rGO nanoparticles utilizing techniques like scanning electron microscopy (SEM)—Energy Dispersive X-ray (EDX) spectroscopy, Micro-Raman spectroscopy, Fourier-transform infrared (FTIR) spectroscopy, and degree of conversion (DC) analysis. 

## 2. Materials and Methods

Ethical approval was acquired before the commencement of the study, and all ethical protocols were firmly followed. The teeth used in the current study were collected from the institute’s orthodontic clinics. 

### 2.1. Fabrication of the EA

The EA was synthesized following the prior recommendations of Ye et al. [24], and the steps of the preparation of EA mentioned in our previous study [25] were precisely replicated.

### 2.2. Addition of rGO Nanoparticles in The Adhesive

The rGO nanoparticles were commercially acquired (Sigma-Aldrich, Taufkirchen, Germany) and then added to the adhesive. The rGO powder was incorporated in 2 mL ethanol inside a microvial and sonicated at 37 °C for 10 min in an ultrasonicator (VWR USC-TH sonicator bath, Tokyo, Japan). rGO powder was mixed with the ethanol solvent of the formulated EA at 0% to produce EA-rGO-0% (control) and at 5 wt.% to yield rGO-5%. To ensure homogenous dispersion of the filler particles, the rGO nanoparticles were mixed in resin and sonicated for 10 min inside an ultrasonic bath (VWR USC-TH sonicator bath, Tokyo, Japan). After sonication, this blend was placed inside an ultrasonic homogenizer (Q500 Sonica) to yield a homogenized mixture at a pulse on/off for one minute at room temperature. To certify that the rGO nanoparticles were evenly dispersed post-storing, the mix was re-homogenized in the same ultrasonic homogenizer after every single use. To protect the notion that precise quantity of rGO nanoparticles were incorporated in the EA, these particles were first weighed up (in milligrams), and their volume was then computed (in milliliters). The subsequent formula was used to calculate weight/volume % (*w/v* %) for the EA group, as suggested formerly by AlFawaz et al. [25]:

*w*/*v*% = weight of solute/volume of solution × 100

The newly synthesized adhesives with and without rGO nanoparticles (EA-rGO-0% and rGO-5%, respectively) were stored at 4 °C and used in our experiments within 14 days of their preparation.

### 2.3. Characterization of the rGO filler Nanoparticles and Adhesive

The characterization of rGO nanoparticles was achieved via multiple investigative techniques. To characterize morphology of the rGO nanoparticles, SEM technique was used. From both adhesive groups (EA-rGO-0% and rGO-5%), five specimens were synthesized and photo-polymerized with a dental curing light source (Curing Light, Eliphar S10; 3M ESPE, St. Paul, MN, USA) with an output of 600 mW·cm^−2^ for 20 s, polymerized from a distance of 10 mm. Before the SEM, the samples were placed on aluminum stubs and then sputtered with a gold layer for 2 min (Baltec SCD sputter, Scotia, NY, USA). The investigation was accomplished at an accelerating voltage of 30 kV utilizing SEM (JEOL, JSM-6513, SEM, Tokyo, Japan). A number of magnifications (selected on the basis of convenience) were utilized to take SEM micrographs of the specimens. To warrant the presence of rGO nanoparticles inside the adhesive, we used SEM-EDX spectroscopy. EDX spectroscopy also helped the current study in exploring the elemental distribution inside the adhesive. Micro-Raman spectroscopy was also executed to analyze rGO nanoparticles. A micro-Raman spectrophotometer (ProRaman-L Analyzer; TSI, Shoreview, MN, USA) with software (Raman reader^®^) was employed to acquire Raman spectra(s). With the help of a 0.9 objective lens, the laser beam was secured, and 600 mW power was used. One minute scan was completed three times for rGO nanoparticles. The particulars of the spectra were attained with a laser beam wavelength of 532 nm, between 1000 and 1700 cm^−1^, with noise filtration.

### 2.4. Preparation of Teeth, Bonding Procedure, and Grouping of Specimens

Sixty single-rooted maxillary second premolars were chosen for our study after carefully examining their anatomical features. The standardization was achieved by excluding the teeth with curved roots and including the teeth with approximately similar root lengths (21 mm ± 1 mm). These teeth were disinfected for 2 days using 0.5% chloramine solution at 4 °C. Post-disinfection, the teeth were cleaned with distilled water for 5 min. The teeth were stored in 1% thymol solution before their use and were utilized within a month after their collection. The anatomical crowns of all the teeth were cut at the cement-enamel junction with a diamond saw (Buehler Isomet 2000 Precision saw, Illinois, IL, USA) to acquire roots with a uniform length of 19 mm. The canals of the attained roots were shaped and prepared using a step-back technique 1 mm short of the radiographic apex. The cleaning and shaping of the canals were achieved via K-files (MANI, Tochigi, Japan) and Gates Glidden drills (MANI) Nos. 2, 3, and 4, correspondingly. Rinsing was achieved with 10 mL of 2.5% sodium hypochlorite (NaOCl) with a 10 mL BD disposable syringe. File No. 35 was designated as the master apical file. The canals were dried using paper points (GapaDent; Zhengzhou Smile Dental Equipment, Zhengzhou, Henan, China). To obturate the canals, gutta-percha (GapaDent) and AH26 sealer (Dentsply DeTrey, Konstanz, Germany) were combined, and the lateral compaction method was used. After 24 h of the obturation, 10 mm of each canal length were prepared using peeso reamers (MANI) of Nos. 4, 3, and 2. Distinctive drills were given by the manufacturer of No. 100 fiber posts (Endolight Post; RTD, St. Egerve, France) to standardize and manage the procedures better. The specimens were then fixed in a condensation silicone mold (Speedex; Coltene/Whaledent, Altstatten, Switzerland), entailing a putty consistency. We utilized 70% ethanol and compressed air to clean and dry the posts, respectively. The specimens were randomly allocated based on the type of adhesive used (EA-rGO-0% or rGO-5%), resulting in 30 samples being assigned to each group. The samples were further separated into groups based on the root canal disinfection technique, including 2.5% NaOCl (control), Photodynamic therapy (PDT) with methylene blue photosensitizer (MBP), and ER-CR-YSGG laser (ECYL) (Biolase^®^, Waterlase I-Plus, MZ8, California, CA, USA), which formed the basis of our six study groups.

Group 1 (EA-NaOCl): The root canal dentin was irrigated with the help of 10 mL of 2.5% NaOCl using a 10-mL BD disposable syringe. Root dentin was treated with EA-rGO-0% adhesive before the cementation of the fiber post. Group 2 (EA-PDT): MBP photosensitizer consisting of 2% aqueous solution was utilized to plug the root canals for 5 min. Light source (diode laser, Handy laser sprint dental; RJ-laser, Winden, Germany) with the power output of 150 mW and wavelength of 638 nm was used to introduce into the canals. The diode laser was used for 2.5 min of irradiation, stopped for 2.5 min, and then irradiation was achieved again for 2.5 min. This protocol was followed to stimulate free radical formation without immensely elevating the destructive temperature. The light was applied as such that it gave 2.5 min of irradiation followed by 2.5 min of stop. To provide a full 360° even radiation, an optical fiber (PACT; Cumdente, Tubingen, Germany) was used. The parameters of the laser encompassed a diameter of 200 mm and taper of 0.03 mm. The root dentin was treated with EA-rGO-0% adhesive before the cementation of the fiber post. Group 3 (EA-ECYL): The ECYL (Er,Cr:YSGG laser) with a frequency of 30 Hz, power of 0.5 W with water/airflow of 25%, with a tip diameter of 275 μm was used. The laser optical fiber was inserted into the full root canal, and irradiation was carried out from apical to the cervical axis in a spiral way with a 2 mm/sec ratio, and irrigation was performed using 2.5% NaOCl. Five cycles of irradiation were accomplished with a time interval of 20 sec in between each cycle, and the roots were cooled off between the cycles. As with the previous two groups, the root dentin was treated with EA-rGO-0% before the cementation of the fiber post. Group 4 (rGO-5%-NaOCl): Irrigation of the root dentin was carried out with 10 mL of 2.5% NaOCl using a 10-mL BD disposable syringe. rGO-5% adhesive was smeared to the root canal dentin preceding fiber post cementation. Group 5 (rGO-5%-PDT): Root canal dentin was treated with PDT as explained in the former section. rGO-5% adhesive was smeared to the root canal dentin before fiber post cementation. Group 6 (rGO-5%-ECYL): Root canal dentin was treated with ECYL as described before. rGO-5% adhesive was smeared to the root canal dentin earlier to fiber post cementation.

Group 1 (EA-NaOCl): The root canal dentin was irrigated with the help of 10 mL of 2.5% NaOCl using a 10-mL BD disposable syringe. Root dentin was treated with EA-rGO-0% adhesive before the cementation of the fiber post. Group 2 (EA-PDT): MBP photosensitizer consisting of 2% aqueous solution was utilized to plug the root canals for 5 min. Light source (diode laser, Handy laser sprint dental; RJ-laser, Winden, Germany) with the power output of 150 mW and wavelength of 638 nm was used to introduce into the canals. The diode laser was used for 2.5 min of irradiation, stopped for 2.5 min, and then irradiation was achieved again for 2.5 min. This protocol was followed to stimulate free radical formation without immensely elevating the destructive temperature. The light was applied as such that it gave 2.5 min of irradiation followed by 2.5 min of stop. To provide a full 360° even radiation, an optical fiber (PACT; Cumdente, Tubingen, Germany) was used. The parameters of the laser encompassed a diameter of 200 mm and taper of 0.03 mm. The root dentin was treated with EA-rGO-0% adhesive before the cementation of the fiber post. Group 3 (EA-ECYL): The ECYL (Er,Cr:YSGG laser) with a frequency of 30 Hz, power of 0.5 W with water/airflow of 25%, with a tip diameter of 275 μm was used. The laser optical fiber was inserted into the full root canal, and irradiation was carried out from apical to the cervical axis in a spiral way with a 2 mm/sec ratio, and irrigation was performed using 2.5% NaOCl. Five cycles of irradiation were accomplished with a time interval of 20 sec in between each cycle, and the roots were cooled off between the cycles. As with the previous two groups, the root dentin was treated with EA-rGO-0% before the cementation of the fiber post. Group 4 (rGO-5%-NaOCl): Irrigation of the root dentin was carried out with 10 mL of 2.5% NaOCl using a 10-mL BD disposable syringe. rGO-5% adhesive was smeared to the root canal dentin preceding fiber post cementation. Group 5 (rGO-5%-PDT): Root canal dentin was treated with PDT as explained in the former section. rGO-5% adhesive was smeared to the root canal dentin before fiber post cementation. Group 6 (rGO-5%-ECYL): Root canal dentin was treated with ECYL as described before. rGO-5% adhesive was smeared to the root canal dentin earlier to fiber post cementation.

### 2.5. Push-out Bond Strength Test and Failure Mode Investigation

The roots containing cemented posts were sectioned vertically with the help of a Low-speed diamond saw (Micracut; Metkon, Bursa, Turkey) operated under water-cooling. This segmenting helped to achieve 0.5 mm thick six serial perpendicular slices from each root sample (three slices each demonstrating coronal and apical post space region). Push-out bond strength was evaluated with the help of a universal testing machine (Lloyd Instruments, Sheffield, UK). Slices gained through cutting were separately attached to the machine. The apical surface of the slices was subjected to loading while facing a cylindrical plunger with a metal rod (1.2 mm and 0.8 mm diameter for the coronal and apical slices) until failure was noticed. The crosshead speed used for this step was 0.5 mm/min. In total, 24 slices (12 each for the coronal and apical region) were exposed to push-out testing. The formula used to estimate push-out bond strength was

r = N/mm^2^

where (N) and (mm^2^) signify the maximum failure load and post segments bonding area, correspondingly. To calculate the thickness of the slices and post segments diameter (from both coronal and apical region), digital calipers were employed, and the formula used for estimating the bonding surface was

Bonding surface area: π (r1 + r2) × (√ (r1 − r2)2 + h2)

where π = 3.14, r1 = radius of the coronal post, r2 = radius of the apical post, and *h* = slice thickness.

The nature of the bond failure was also investigated in our study for the cervical and apical root dentin with the help of a digital microscope (Hirox KH 7700, Tokyo, Japan). The failure modes were classified into adhesive-post, adhesive-dentin, and mixed types.

### 2.6. SEM Investigation of The Root Dentin Adhesive Interface

SEM was used to assess the root dentin interface of ten segmented root dentin samples from each group. The samples were fixed on aluminum stubs and sputtered with a gold layer and witnessed under SEM (JEOL, JSM-6513, SEM, Tokyo, Japan). The accelerating voltage of the SEM was 30 kV whereas, different magnifications (based on convenience) were utilized.

### 2.7. FTIR Spectroscopy, DC Analysis, and Rheometry

The specimen size was 10 × 2 × 2 mm. FTIR spectroscopy was employed to estimate the DC of both the adhesive groups (EA-rGO-0% and rGO-5%). The steps of the FTIR spectroscopy mentioned in our previous study [26] were precisely replicated. Using a previous recognized method [27], C-C aromatic reference peaks (1607 cm^−1^) and C = C (aliphatic) absorbance peaks (1638 cm^−1^) were acquired. The transformation rates for the adhesives were estimated using the ratios of C=C and C-C absorbance strengths (% of unreacted double bonds) pre-and post-curing by means of the following equation, recommended earlier by Al-Hamdan et al. [26]:

DC = [1 − (C aliphatic/C aromatic)/(U aliphatic/U aromatic)] × 100%

where, C aliphatic is 1638 cm^−1^ absorption peak after curing, C aromatic is 1607 cm^−1^ absorption peak after curing, U-aliphatic is 1638 cm^−1^ absorption peak before curing and U-aromatic is 1607 cm^−1^ absorption peak before curing.

Utilizing an MCR-72 rheometer (Anton Paar, Graz, Austria), the rheological properties of the composites were examined at rotation mode in a frequency sweep predetermined state of 8 mm parallel plate, 0.25 mm opening, and over an array of angular frequencies stretching between 0.1–100 rad/s at 25 °C.

### 2.8. Statistical Analysis

The statistical analysis was carried out for the push-out bond strength and DC analysis values utilizing SPSS-20.0 (IBM, Chicago, IL, USA). Mean and standard deviation values were calculated, and the ANOVA and post hoc multiple comparison tests were utilized. The level of significance was set at 1%.

## 3. Results

### 3.1. rGO Filler Nanoparticles Characterization Outcomes

**Figure 1:**
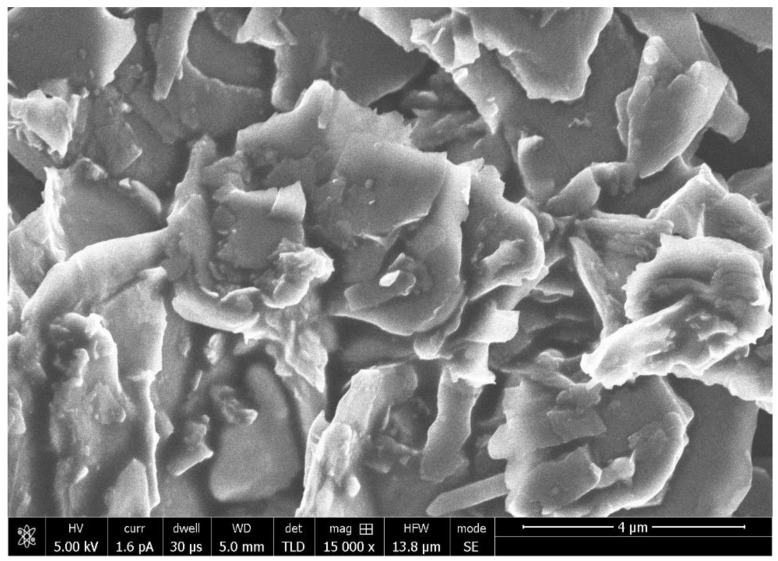
SEM image of graphene oxide. Graphene oxide showing variable size flakes with 1–2 nm thickness. These nanoparticles on SEM shows uniform and tight agglomerations for graphene oxide flakes.

**Figure 2:**
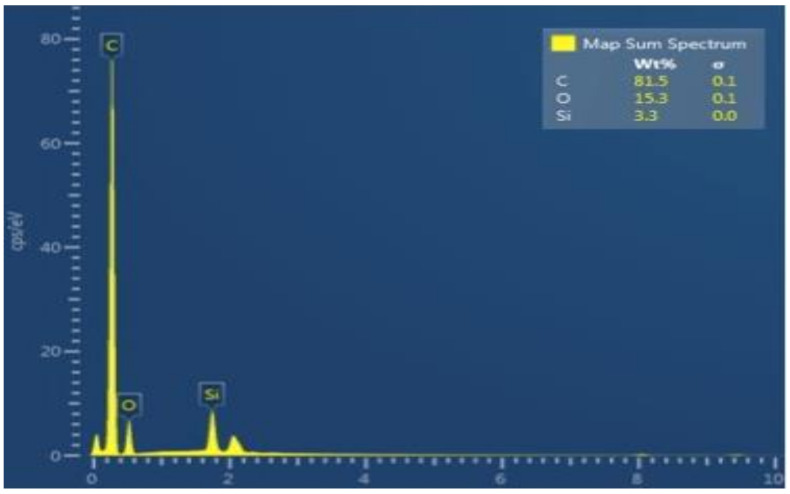
The EDX line analysis (rGO 5 wt%) adhesive showing evidence of high carbon percentage (81.5%) along with Si dispersion in the experimental adhesive.

**Figure 3:**
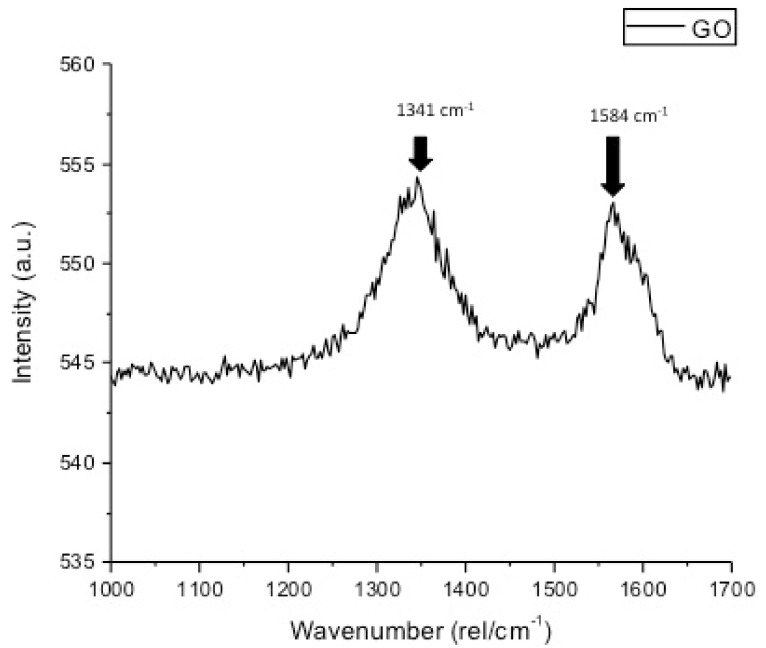
Raman spectra of (A) GO nanoparticles showing characteristic D and G bands which are two prominent peaks at 1341 and 1584 cm^−1^. Usually, the G mode is assigned to the vibration of sp2-hybridized carbon, while the D mode is related to the sp3-hybridized carbon and deficiencies relevant with grain boundaries and vacancies in GO.

The representative SEM micrograph demonstrated flake-shaped rGO nanoparticles with coarse ends, which were 1–2 nm in size (Figure 1). The EDX analysis verified the highest percentage of carbon (C) (81.5%) along with other ions for the rGO-5% group (Figure 2). The existence of C in the EDX mapping warrants the incidence of graphene. The representative Raman spectra of rGO-5% nanoparticles are shown in Figure 3. Two Raman bands, D and G, were witnessed at 1341 cm^−1^ and 1584 cm^−1^, correspondingly. Characteristically, the D band represents sp3-hybridized carbon and deficits associated with grain boundaries and vacancies in rGO, while the G band signifies the vibration of sp2-hybridized carbon.

### 3.2. Adhesive-Dentin Interface, Push-out Bond Strength, and Interfacial Failure Analysis Results

**Figure 4:**
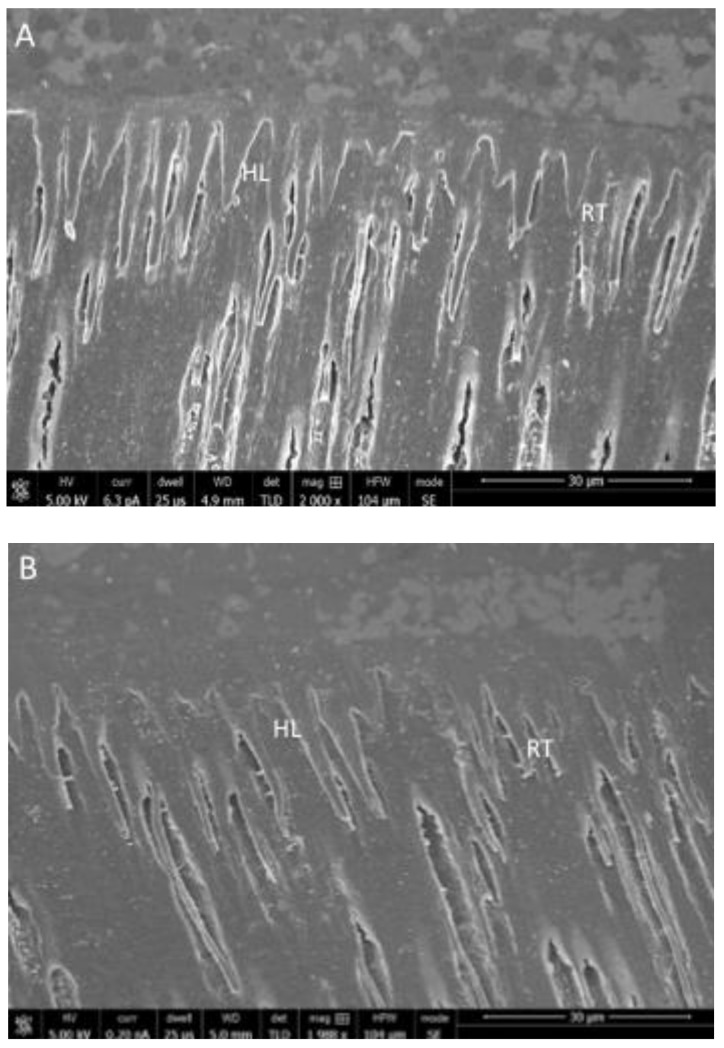

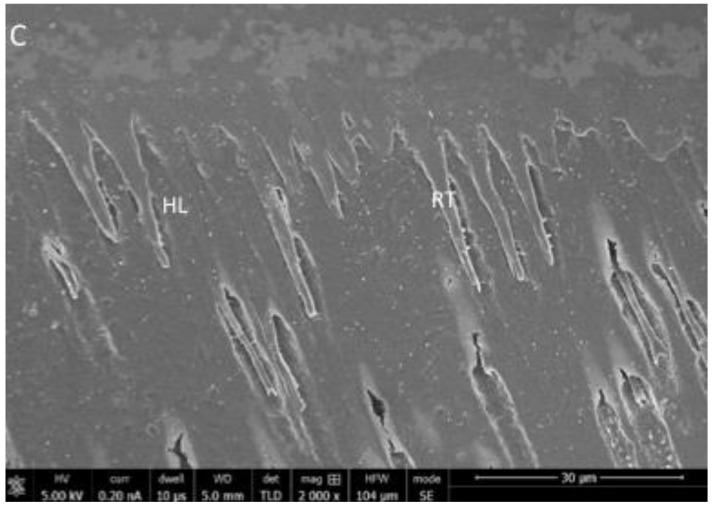
SEM images of bonded dentin specimen (**A**) rGO-5%- NaOCl group specimens showing classic hybrid layer (HL) formation with high number of resin tags in dentin. (**B**) rGO-5%-PDT group specimens showing standard HL formation with average number of resin tags (**C**) rGO-5%-ECYL group specimens displaying lower than average resin tags on the dentin surface at the interface.

The representative SEM micrographs illustrating resin tag formation for rGO-5% adhesive are presented in Figure 4A–C. A high number of resin tags of varying depths were observed for rGO-5%-NaOCl with classic hybrid layer (HL) formation (Figure 4A). For rGO-5%-PDT, standard HL formation and fewer resin tags (compared to rGO-5%-NaOCl) were observed (Figure 4B). The rGO-5%-ECYL presented only a few resin tags on the adhesive-dentin interface (Figure 4C). A similar pattern was observed for the resin tags seen for EA-rGO-0% adhesive, where the number of tags was high for EA-rGO-NaOCl, followed by EA-rGO-PDT, and the least number of tags were observed for EA-rGO-ECYL.

The outcomes of the push-out bond strength (presented as means ± standard deviations) are presented in Table 1. The highest push-out bond strength was attained for EA-rGO-0%-NaOCl (9.40 ± 1.31) followed by rGO-5%-NaOCl (9.13 ± 1.78). The next greatest values were observed for EA-rGO-0%-PDT (8.77 ± 1.30) trailed by rGO-5%-PDT (8.26 ± 1.25). The lowest values were appreciated for ECYL treated groups (EA-rGO-0%-ECYL: 6.63 ± 1.26, rGO-5%-ECYL: 7.14 ± 1.30). On statistical comparison, all the intra-group differences were not statistically significant (*p* > 0.01). The inter-group comparisons were statistically significant (*p* < 0.01) when EA-rGO-0%-NaOCl and EA-rGO-0%-PDT values were compared with EA-rGO-0%-ECYL values. The inter-group comparisons were also statistically significant (*p* < 0.01) when rGO-5%-NaOCl and rGO-5%-PDT values were compared with rGO-5%-ECYL values.

The percentages of interfacial failure types observed among different study groups of our study are presented in Table 2. The bulk of the failures in our study were of adhesive-dentin type. For the NaOCl treated cervical root segments, 80% failures were of the adhesive-dentin kind, and 20% were mixed. For the PDT treated cervical root segments, 80% failures were of adhesive-dentin type whereas, 10% failures each were of adhesive-post and mixed type. For the ECYL treated cervical root segments, similar numbers were observed as PDT treated cervical root segments. Concerning apical root segments treated with NaOCl and ECYL, 100% of the failures were adhesive-dentin type. For the PDT treated apical root segments, 80% of failures were adhesive-dentin type, whereas 20% mixed failures were observed.

### 3.3. FTIR Spectroscopy and DC Analysis Outcomes

**Figure 5:**
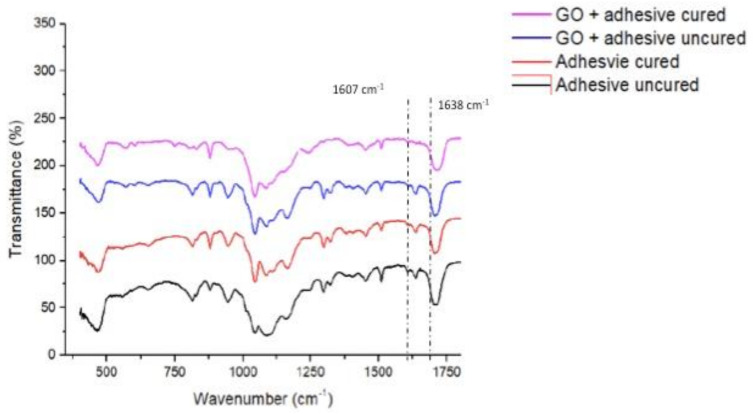
FT-IR spectrum of uncured and cured resin adhesives containing graphene oxide nanoparticles, and control (no nanoparticles). The degree of conversion was estimated from 1607 cm^−1^ and 1638 cm^−1^ wavenumber.

The characteristic FTIR spectra after the mixing of rGO nanoparticles (for cured and uncured adhesive) are shown in Figure 5. The DC was projected from 1607 cm^−1^ and 1638 cm^−1^ wavenumbers. EA-rGO-0% presented with a higher DC (57.8 ± 8.7) as compared to rGO-5% (51.3 ± 6.6). No statistically significant results (*p* < 0.01) were perceived when the DC values of these two groups were compared (Table 3).

### 3.4. Rheological Properties Analysis Results

**Figure 6:**
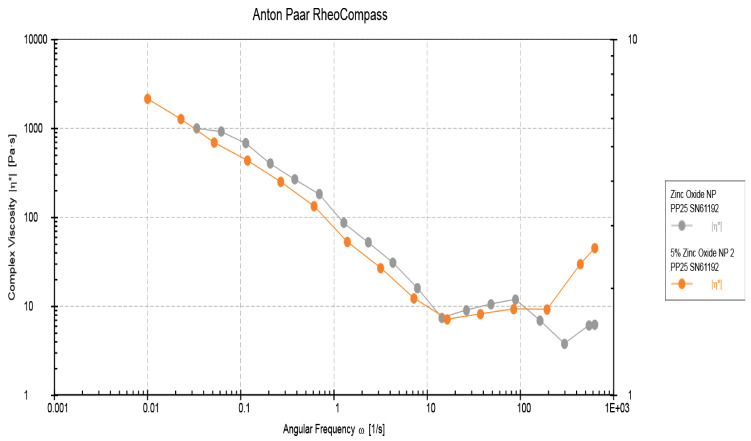
Rheological properties of the experimental control (EA-0% rGO) and rGO 5% adhesives. Complex viscosity vs. angular frequency at 0.001 to 1000 rads/s.

The complex viscosities versus angular frequency of the two adhesive groups (EA-rGO-0% and rGO-5%) at 0.001 to 1000 rads/s are shown in Figure 6. The two adhesive groups demonstrated reduced viscosity as the frequency increased. Therefore, it can be established that our two adhesive samples presented assertive non-Newtonian behavior (shear-thinning, that is, with growing shear rate, reduced viscosity was observed, which is called pseudo-plasticity). Lower complex viscosity values were observed for rGO-5% compared with EA-rGO-0% at various equivalent angular frequencies (Figure 6). This demonstrates that the addition of rGO-5% nanoparticles led to an altered fluidity of the resin/filler suspension; nevertheless, a Newtonian plateau was not perceived (even at the lowest frequencies). The reduced viscosity shown by the rGO-5% group could be linked with the plasticizing behavior of rGO sheets by augmenting the mobility of polymer chains to slip over each other. Although the viscosities shown by the EA-rGO-0% group were higher than the rGO-5% group, they were still comparable. Hence, it can be concluded that the incorporation of rGO-5% nanoparticles led to a reduction in the viscosity; however, it did not sustain, and at high frequencies, an intersection was detected between the two viscosities (Figure 6).

## 4. Discussion

Based on our study results, we can partially accept the hypothesis that the addition of rGO-5% nanoparticles improved the adhesive’s dentin interaction and bond strength. The hypothesis is also partially rejected as the DC of the adhesive decreased, and lower complex viscosity of the rGO-5% group were observed when matched with the EA-rGO-0% group. The clinical success of a restoration is particularly reliant on the adhesive properties, and a compromised adhesive-dentin bond could cause micro-leakage, development of secondary caries, and eventually, failure of the restoration [28]. Previously, the addition of various nano-inorganic fillers in the adhesive has yielded very positive outcomes [29,30]. Notably, the incorporation of GO and rGO filler nanoparticles inside the composite resin and adhesive has been shown to improve the adhesive’s mechanical properties in the past [22,25]. Considering the advantageous properties of rGO filler nanoparticles, we also wanted to explore the effect of their addition on various properties of the adhesive. Previously, Wagner et al. reported that filler content’s addition > 10 wt.% could negatively impact the bond strength of the adhesives [31]. Considering this recommendation, we also used only 5 wt.% rGO filler nanoparticles in our study.

The rGO nanoparticles, when observed on the SEM, revealed flake-shaped morphology, and this finding is consistent with several previous studies [25,32]. In general, the reduction process to yield GO nanoparticles produces flake-shaped sharp-edged sheets due to the presence of oxygen groups in the oxide sheets [33]. The Micro-Raman representative spectra of rGO-5% adhesive demonstrated two vibrational bands; the D band was seen at 1341 cm^−1^ whereas the G band was perceived at 1584 cm^−1^, and this particular finding was similar to a previous study [25]. For the materials having graphite, the observation of two bands is common as they are created due to the shift in energy caused by the laser excitation [34]. Concerning resin tags, rGO-5% demonstrated comparable resin tags (Figure 4A–C) to EA-rGO-0%. However, NaOCl treatment was less severe than PDT, which was less destructive than ECYL treatment, demonstrated by reducing the number of tags seen after these three treatments, respectively. The credible reason for this outcome could be that we used ECYL with a high frequency (30 Hz). Wang et al. previously reported that using a laser with high frequency (25–30 Hz) could cause cracking and result in the negotiated structural integrity of dentin [35]. Another probable reason for this finding could be that the use of laser ablation causes fusion of dentinal collagen fibers, giving less space for the penetration of the adhesive and formation of resin tags, as explained earlier by Ceballo et al. [36]. These resin tags seen for rGO-5% were of variable depths (penetration depths were not recorded in this study), but resin tags depth neither significantly impact the intactness of the adhesive nor affects its bond strength, as proposed earlier by Anchieta et al. [37].

Push out bond strength is a consistent and practical method to test the adaptation strength of a material to its neighboring root dentin [38]. Our push-out bond strength test results demonstrated higher values for NaOCl conditioned dentin followed by PDT and then ECYL. Previously, Al Jeaidi explained that the canals treated with NaOCl revealed higher push-out bond strength of fiber-reinforced composite post than PDT treated samples [39], and our results are in harmony with this study. This was probably observed due to the cationic nature of MBP, which can bind to calcium and phosphate ions of the dentin, disturbing their ratio causing precipitation in radicular dentin, therefore, resulting in a compromised dentin adhesive interaction [40]. Our results are also in arrangement with Alonaizan et al. who demonstrated that the PDT group in their study revealed higher push-out bond strength values when matched with ECYL group [41]. A plausible reason to explain this finding could be that high-frequency laser ablation creates a thermal effect, which affects dentinal walls negatively, leading to a decreased push out bond strength [42]. PDT on the other hand is less invasive (compared with ECYL) [41], and a similar previous study demonstrated that the bond strength values of PDT treated dentin were higher that laser conditioned dentin [43].

Concerning interfacial failure types, most of the failures witnessed in our study were of the adhesive-dentin type, followed by mixed, and a few were adhesive-post type. These findings conformed with several previous studies [44,45]. Adhesive-post and mixed failures were primarily observed in the cervical third of the root, and this finding matched the results of Vohra et al. [44]. The reason for observing higher push-out bond strength in the cervical third of the root as opposed to the apical region could be attributed to impartial polymerization or inconsistent positioning of the posts [46].

DC of the two adhesive groups was also evaluated in our study, and although the results demonstrated comparable DC for both adhesive groups, still, higher DC values for EA-rGO-0% adhesive were seen compared with rGO-5% adhesive. Earlier, Bin-Shuwaish et al. also reported an inverse positive linear relationship between increasing GO content and DC [32], and our results agree with their findings. Aside from rGO, researchers have also previously incorporated various other inorganic fillers like hydroxyapatite and silica in the adhesive and reported a similar trend (DC decreases linearly with the increasing filler content) [27,47]. A logical reason to explain this could be the nature of the inorganic fillers, which are opaque to the light, possibly hindering the adequate conversion of monomers into polymers, thus, resulting in low DC [48].

In relation to the rheological properties, two adhesive groups demonstrated reduced viscosity as the frequency increased, and rGO-5% demonstrated lower complex viscosity values were when matched with EA-rGO-0% at various equivalent angular frequencies. Comparable findings were previously conveyed by Alrahlah et al. who revealed that the decrease in viscosity could be linked to increasing rGO filler content [22]. This reduction in the viscosity could be linked with the plasticizing nature of rGO sheets, which could have enhanced polymer chains’ movement to slide past each other. Subsequently, the viscosity of the adhesive gets reduced [49].

The findings of the current study, although encouraging, should be cautiously interpreted. The rGO-5% adhesive demonstrated comparable push-out bond strength and resin tag formation to the control group; however, a decreased DC was observed. As poor DC influences durability and water sorption of adhesive, the properties of adhesives with variable aging techniques should be explored in future studies. Moreover, studies aimed at exploring the effect of lower concentration of rGO nanoparticles in the adhesive should be carried out to further investigate this area.

## 5. Conclusions

The rGO-5% adhesive presented comparable push-out bond strength and dentin interaction that matched the control group. However, a lower DC was detected for rGO-5% adhesive. The push-out bond strength was highest after NaOCl conditioning, followed by PDT and ECYL treatment for both adhesive groups. rGO could prove to be a promising filler in dentin adhesives, and future work is encouraged to probe the effect of various concentrations of rGO nanoparticles on the adhesive’s properties.

## Figures and Tables

**Table 1:** Push-out bond strength of surface treated adhesive bonded dentin specimens in the study (values in MPa).

Adhesive	NaOCl	PDT	ECYL	*p* Value
EA-rGO 0%	9.40 ± 1.31 ^A a^	8.77 ± 1.30 ^A a^	6.63 ± 1.26 ^A b^	<0.01
rGO-5%	9.13 ± 1.78 ^A a^	8.26 ± 1.25 ^A a^	7.14 ± 1.30 ^A b^	<0.01

NaOCl: Sodium hypochlorite, PDT: Photodynamic therapy, ECYL: Er:Cr:YSGG laser, EA: Experimental adhesive. Dissimilar uppercase alphabets in same column denote statistical difference. Dissimilar lowercase alphabets in the same row denote statistical significant difference.

**Table 2:** Percentage distribution of interfacial failure types among the study groups.

		Type of Failure		
Study Group	Root Segment	Adhesive-Post	Adhesive-Dentin	Mixed
NaOCl	Cervical	0	80	20
	Apical	0	100	0
PDT	Cervical	10	80	10
	Apical	0	80	20
ECYL	Cervical	10	80	10
	Apical	0	100	0

**Table 3:** Degree of conversion (DC) displayed by EA-rGO-0% and rGO-5% adhesive.

Group	DC (Mean ± SD)	Tukey (*p* < 0.01)
EA-rGO-0%	57.8 ± 8.7	A
rGO-5%	51.3 ± 6.6	A

rGO: Reduced Graphene oxide, EA: Experimental adhesive (control). Similar uppercase letters indicate no statistical significance.

## Data Availability

The data will be available from the corresponding author on contact.
